# Clinical application of chest CT image screening combined with nanopore targeted sequencing in improving the diagnosis rate of pulmonary tuberculosis patients

**DOI:** 10.3389/fpubh.2026.1826208

**Published:** 2026-07-23

**Authors:** Jiayuan Ye, Dong Lv, Yifei Tu, Shanshan Zhao, Shanshan Yuan, Xiang Xu, Xiaotong Ding, Zhouxiao Wu, Yuxiang Xu, Dongdong Zhao, Yilian Xie

**Affiliations:** 1 Department of Infectious Diseases, Shangyu People's Hospital of Shaoxing, Shaoxing University, Shaoxing, Zhejiang, China; 2 Health Science Center, Ningbo University, Ningbo, Zhejiang, China; 3 Department of Respiratory, Shangyu People's Hospital of Shaoxing, Shaoxing University, Shaoxing, Zhejiang, China; 4 Department of Radiology, Shangyu People's Hospital of Shaoxing, Shaoxing University, Shaoxing, Zhejiang, China; 5 Department of Clinical Laboratory, Shangyu People's Hospital of Shaoxing, Shaoxing University, Shaoxing, Zhejiang, China; 6 Zhejiang Key Laboratory of Digital Technology in Medical Diagnostics, Hangzhou, Zhejiang, China; 7 Department of General Medicine, Shangyu People's Hospital of Shaoxing, Shaoxing University, Shaoxing, Zhejiang, China; 8 Department of Medical, Shangyu People's Hospital of Shaoxing, Shaoxing University, Shaoxing, Zhejiang, China; 9 Department of Infectious Diseases, Sir Run Run Shaw Hospital, Zhejiang University School of Medicine, Hangzhou, Zhejiang, China; 10 Department of Infectious Diseases, The First Affiliated Hospital of Ningbo University, Ningbo, Zhejiang, China

**Keywords:** bronchoalveolar lavage fluid, chest CT image screening, clinical diagnosis, nanopore targeted sequencing, pulmonary tuberculosis

## Abstract

**Background:**

How to increase the diagnosis rate of pulmonary tuberculosis (PTB) has always been a major challenge in medicine. Compared with conventional microbiological tests (CMTs), nanopore targeted sequencing (NTS) is undoubtedly a faster and more effective diagnostic method. This study explores the feasibility of combining chest CT imaging screening with NTS to improve PTB diagnosis.

**Method:**

This retrospective observational study was conducted from March 2023 to December 2024, enrolling 380 patients from a single center who underwent bronchoalveolar lavage fluid (BALF) analysis and NTS testing. We compared the diagnostic accuracy of radiologists with varying experience levels to determine the most suitable preliminary screening protocol for PTB. Additionally, we compared NTS results with CMTs (including mycobacterial culture, acid-fast bacilli smear and Xpert MTB/RIF) to evaluate differences in diagnostic performance between NTS and CMTs.

**Results:**

We conducted a horizontal comparison of PTB diagnostic accuracy among three radiologists with different levels of experience. The most experienced radiologist had the lowest missed diagnosis rate. When combining cases suspected by all three radiologists (202 cases), the missed diagnosis rate dropped to as low as 9.09% (27.27% [95% CI: 16.77–40.70%] vs. 30.91% [95% CI: 19.73–44.51%] vs. 16.36% [95% CI: 8.38–29.82%] vs. 9.09% [95% CI: 3.80–19.84%], *p* = 0.019). Furthermore, we analyzed the diagnostic performance of NTS and CMTs in PTB patients and found that NTS had significantly higher sensitivity than Xpert MTB/RIF, mycobacterial culture, and acid-fast bacilli smear (89.09% [95% CI: 78.17–95.02%] vs. 78.18% [95% CI: 65.44–87.12%] vs. 23.64% [95% CI: 13.99–36.99%] vs. 16.36% [95% CI: 8.38–28.82%], *p* < 0.001). ROC analysis demonstrated that incorporating NTS into the testing of suspected PTB specimens significantly improved diagnostic capability (AUC = 0.990, 95% CI: 0.968–1.000).

**Conclusion:**

Radiologists’ experience in PTB imaging diagnosis can reduce missed diagnoses. Additionally, combining NTS with CMTs helps improve early diagnosis in suspected PTB patients. The combined use of NTS and Xpert MTB/RIF yielded the highest diagnostic accuracy (AUC 0.990).

## 1 Introduction

Tuberculosis (TB) is an airborne infectious disease caused by *Mycobacterium tuberculosis* (MTB). According to global statistics, approximately one-quarter of the world’s population is infected with MTB (1). With the decline in COVID-19 mortality rates, it is alarming that tuberculosis (TB) has re-emerged as the leading cause of death from infectious diseases worldwide (2). Among the 30 countries with the highest TB burden, China ranks third globally in the estimated number of TB cases, contributing 7.1% to the global total, following India (27%) and Indonesia (10%) (3).

Delays in seeking medical care for TB patients can lead to the transmission of MTB, exacerbate patient suffering and economic burdens, and increase morbidity and mortality rates (4, 5). Currently, the initial diagnosis of pulmonary Tuberculosis (PTB) faces multiple challenges. Although chest CT imaging is more sensitive than X-rays (6), its nonspecific features make interpretation highly dependent on radiologists’ experience (7), leading to frequent misdiagnosis or missed diagnosis. Meanwhile, among conventional microbiological tests (CMTs) for MTB, acid-fast bacillus (AFB) smear offers rapid, simple, and low-cost detection but suffers from low pathogen positivity rates, whereas mycobacterial culture methods are time-consuming (8), making both unsuitable for rapid clinical diagnosis.

The emergence of molecular methods has reduced MTB diagnosis time to a few hours. For example, the Xpert MTB/Rifampicin (RIF) assay can reduce the likelihood of human-to-human transmission as well as TB-related morbidity and mortality (9). However, many studies have found that its sensitivity remains suboptimal for samples with low bacterial loads, failing to meet the diagnostic needs of PTB (10). The advent of nanopore targeted sequencing (NTS) technology has largely compensated for this limitation. Targeted amplification technology offers advantages such as high specificity, low sample requirements, relatively simple laboratory procedures, and lower costs. Among these, nanopore sequencing enables real-time bioinformatics analysis during sequencing, thereby shortening experimental time (11). The NTS method combines nanopore sequencing technology with targeted amplification techniques, such as those targeting the bacterial 16S rRNA gene and the mycobacterial rpoB gene. NTS shows promise in overcoming the limitations of culture, polymerase chain reaction (PCR), and metagenomic next-generation sequencing (mNGS) (12). Additionally, preliminary studies have demonstrated that NTS and Xpert MTB/RIF are complementary in diagnosing PTB (13).

In this context, building upon our previous work that demonstrated the utility of Nanopore Targeted Sequencing (NTS) for broad pathogen detection in bronchoalveolar lavage fluid (BALF) from patients with pulmonary infections (14), we conducted a retrospective analysis of 380 patients from a single tertiary medical center between March 2023 and December 2024. All patients underwent BALF collection and NTS testing. Unlike the prior study which evaluated NTS across a spectrum of pathogens, the present investigation shifts focus specifically to pulmonary tuberculosis (PTB), integrating radiologist-led chest CT screening with NTS technology. This study therefore aimed to evaluate both the impact of radiologists’ experience on diagnostic accuracy and the added value of combining nanopore targeted sequencing (NTS) with conventional testing for *Mycobacterium tuberculosis* detection, thereby assessing the feasibility and clinical utility of this strategy.

## 2 Materials and methods

**Figure 1:**
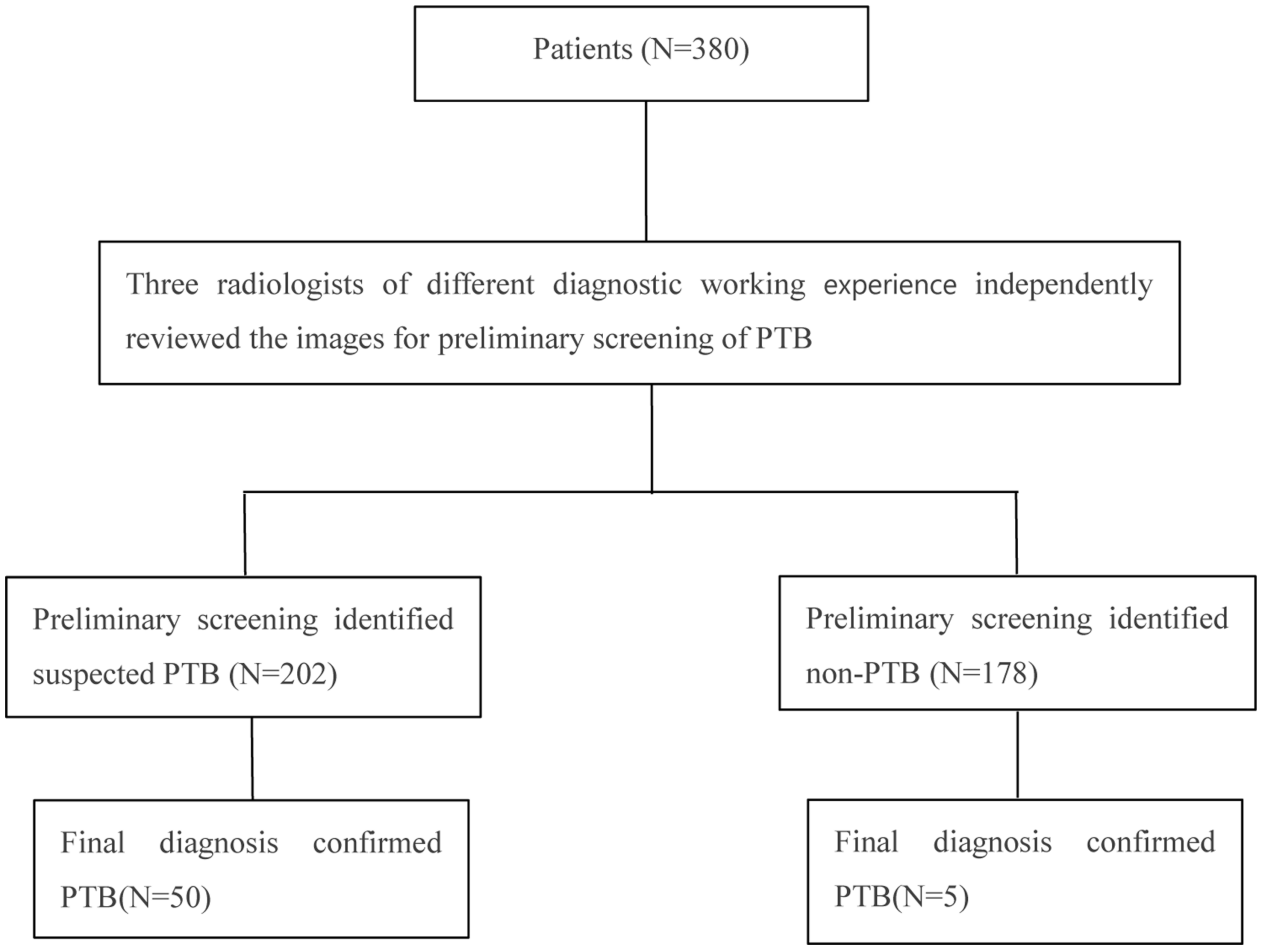
Flow-chart of the study samples.

This study was conducted at Shangyu People’s Hospital of Shaoxing, Zhejiang Province, China, from March 2023 to December 2024. A total of 380 patients were finally included (Figure 1). All patients underwent bronchoscopy, with bronchoalveolar lavage fluid (BALF) collected and subjected to NTS and CMTs testing.

### 2.1 Inclusion and exclusion criteria

Inclusion criteria were as follows:

1. Patients of any age or gender.
2. Specific diagnostic criteria for pulmonary infection, including new or worsening focal/diffuse infiltrates on chest CT imaging, combined with clinical manifestations such as new-onset fever, cough, increased sputum production, shortness of breath, fatigue, or hemoptysis.
3. Collection of BALF.
4. BALF tested with NTS, Xpert MTB/RIF, AFB, and mycobacterial culture.
5. Patients or their legal guardians provided informed consent.

Exclusion criteria were as follows:

1. Cases with incomplete data.
2. Sample issues: Insufficient sample volume or failure to meet standard requirements.

The study was approved by the Ethics Committee of Shangyu People’s Hospital of Shaoxing (2025–017-01). Written informed consent was obtained from the individual(s) for the publication of any potentially identifiable images or data included in this article.

### 2.2 Reference standard for pulmonary tuberculosis diagnosis

The final clinical diagnosis for each participant served as the reference standard for evaluating test sensitivity and specificity. Diagnoses were determined at discharge through consensus by a medical team comprising two infectious disease specialists and one radiologist. In cases of disagreement, a panel of three independent experts convened to reach a definitive diagnosis. The assessment integrated clinical manifestations, CMTs, NTS findings, and other relevant diagnostic evidence, based on the “Diagnosis of Pulmonary Tuberculosis - Health Industry Standard of the People’s Republic of China (WS 288–2017)” (15). According to this standard, patients were classified into the following categories:

1. Confirmed TB cases: Positive etiological tests (smear, culture, or molecular biology) with clinical diagnosis.
2. Clinically diagnosed TB cases: Abnormal chest imaging consistent with TB after excluding other pulmonary diseases, along with TB-compatible symptoms/signs or positive immunologic TB tests.
3. Suspected TB cases: Only abnormal chest imaging suggestive of TB.
4. Non-TB cases: TB was ruled out after differential diagnosis (15).

### 2.3 Imaging-based preliminary screening

The chest CT images of all patients were independently reviewed by three radiologists with 8, 15, and 22 years of experience in thoracic imaging, respectively, each providing their own diagnostic assessment for initial PTB screening. Each radiologist classified patients into “suspected TB” or “TB-excluded” groups.

If all three radiologists excluded TB, the patient was assigned to the “TB-excluded” group (*n* = 178).

If at least one radiologist suspected TB, the patient was assigned to the “suspected TB” group (*n* = 202).

### 2.4 Sample collection and laboratory testing

All patients underwent bronchoscopy. BALF was collected from lesion sites identified on chest CT imaging and further tested using CMTs and NTS.

#### 2.4.1 CMTs testing

Routine microbiological tests were performed on BALF samples. AFB, mycobacterial culture, and Xpert MTB/RIF were employed.

Mycobacterial culture: According to the “Regulations on Clinical Trial Operation of Pathogen Detection in National Clinical Microbiology Laboratories (16)”, all samples underwent routine analysis. BALF was concentrated by centrifugation at 3,000 rpm for 10 min, and then inoculated onto blood agar plates, MacConkey agar plates, and chocolate agar plates, which were cultured at 35 °C with 5% CO2 for 7 days. For mycobacterial cultures, samples were inoculated into Roche medium and cultured for 42 days.

AFB smear: Acid-fast staining was performed using the Ziehl-Neelsen method. Staining analysis was conducted using the BR-101 fully automated tuberculosis-acid-fast staining instrument (Qingdao Lance Biotechnology Co., Ltd.). Subsequently, smear readings were performed using a slide viewer (Ningbo Shunyu automatic smear reader). Positive samples were rechecked at 1000x magnification under an Olympus optical microscope.

Xpert MTB/RIF: Mix the centrifuged lavage fluid sample with 2 mL of sample processing solution. After vortexing for 15 s, incubate the mixture at room temperature for 15 min. Then, aspirate the liquefied sample into the dedicated sterile transfer pipette of the Xpert MTB/RIF test cartridge; open the test cartridge and slowly add the processed sample into the sample well of the cartridge, and close the test cartridge for automated testing. The system automatically reads the MTB test results within 2 h (Xpert MTB/RIF semi-nested fully automated real-time fluorescence quantitative PCR detection technology 301-3300-ZH-CN).

#### 2.4.2 NTS testing

BALF samples (>5 mL) were collected and immediately transported on dry ice to Hangzhou Dean Medical Laboratory for Nanopore targeted sequencing (NTS). The detailed protocol—including sample liquefaction, nucleic acid extraction, targeted amplification, library construction, and sequencing on the GridION MK1 platform—has been described in our previous methodological study (14). Briefly, after DNA extraction using a magnetic bead-based automatic extractor, a multiplex PCR panel targeting 354 respiratory pathogens (Supplementary Table 1) was used for library preparation. Sequencing was performed on a MinION flow cell (R9.4.1) with real-time basecalling performed by MinKnow software. For a complete step-by-step description, readers are referred to Supplementary material 1.

### 2.5 Interpretation of NTS results

The following criteria were applied sequentially to determine positive and negative results.

Quality control (QC). A sample was considered to have passed QC only if both of the following conditions were met: (i) total sequencing data volume reached ≥30 Mb, and (ii) the number of reads mapping to the internal control (Lactococcus spp., spiked at a known concentration during sample processing) exceeded 100. Samples failing QC were excluded from further analysis.

Taxonomic assignment. Species identification was performed by aligning all QC-passed reads against a curated reference database using BLAST (Basic Local Alignment Search Tool). A read was assigned to a specific species when both of the following thresholds were satisfied: ≥80% of the matched database sequences belonged to that species (identity concordance), and the alignment coverage was ≥85% of the reference sequence length. Reads that did not meet both criteria were discarded.

Relative abundance filtering. For each species that received at least one assigned read, its relative abundance was calculated as the percentage of species-specific reads out of the total number of reads in the same sequencing batch. Any species with a relative abundance <0.5% was considered potential cross-contamination (e.g., from index swapping or reagent carryover) and was excluded from the final positive list, as recommended in reference (13, 17, 18).

#### 2.5.1 Negative control validation

Each sequencing batch included at least one negative control sample (nuclease-free water processed identically to clinical specimens). For any microorganism that was detected in both a clinical sample and its corresponding batch negative control, the following decontamination rule was applied:

The microbe was retained as a true positive only if the read count in the clinical sample exceeded five times the read count in the negative control.

If the ratio was ≤5, the microbe was considered non-pathogenic environmental or reagent contamination and was discarded from the final result.

This 5-fold cutoff was empirically established from historical negative control sequencing runs to minimize false-positive reporting while preserving sensitivity for genuine low-abundance pathogens.

### 2.6 Statistical analysis

SPSS 25.0 (IBM, Chicago, IL, USA) was used for statistical analyses and ROC curve generation. Chi-square tests compared the sensitivity and specificity of NTS versus CMTs, using the final clinical diagnosis as the reference. All tests were two-tailed, with *p* < 0.05 considered statistically significant. ROC curve, Venn diagrams and bubble plots were generated using R software (Version 4.2.2).

## 3 Results

### 3.1 Basic characteristics of patients

A total of 380 patients from one hospital were included in this study. Among them, 202 suspected PTB cases were analyzed in detail, with 50 (24.75%) ultimately confirmed as PTB and 152 (75.25%) excluded from the PTB diagnosis. Baseline analysis of these two groups revealed no statistically significant differences in gender, symptoms, erythrocyte sedimentation rate (ESR), C-reactive protein (CRP), procalcitonin (PCT), comorbidities, pleural thickening, or lymph node calcification, except that PTB patients tended to be older (Table 1). Among the remaining 178 patients initially screened as non-TB cases, five were eventually diagnosed with PTB. We analyzed the symptoms, comorbidities, and chest CT imaging characteristics of these five patients in detail (Table 2).

**Table 1:** Baseline characteristics of confirmed PTB and excluded non-TB patients among the 202 cases.

TB	No	Yes	*p*-value
N	152	50	
CRP (mg/L)	43.15 ± 75.60	31.18 ± 42.78	0.299
PCT (ng/ml)	1.79 ± 10.20	0.10 ± 0.17	0.315
ESR. MMH.	31.62 ± 32.86	22.33 ± 25.43	0.121
Age	56.88 ± 15.64	50.40 ± 20.09	0.019
Sex			0.254
Male	96 (63.16%)	36 (72.00%)	
Female	56 (36.84%)	14 (28.00%)	
Sputum or hemoptysis			0.291
No	69 (45.39%)	27 (54.00%)	
Yes	83 (54.61%)	23 (46.00%)	
Fever			0.513
No	118 (77.63%)	41 (82.00%)	
Yes	34 (22.37%)	9 (18.00%)	
Chest pain			0.359
No	145 (95.39%)	46 (92.00%)	
Yes	7 (4.61%)	4 (8.00%)	
Fatigue/night sweats			0.424
No	149 (98.03%)	48 (96.00%)	
Yes	3 (1.97%)	2 (4.00%)	
Chest tightness			0.562
No	139 (91.45%)	47 (94.00%)	
Yes	13 (8.55%)	3 (6.00%)	
Asymptomatic			0.54
No	116 (76.32%)	36 (72.00%)	
Yes	36 (23.68%)	14 (28.00%)	
Bronchiectasis			0.287
No	135 (88.82%)	47 (94.00%)	
Yes	17 (11.18%)	3 (6.00%)	
Diabetes mellitus			0.53
No	141 (92.76%)	45 (90.00%)	
Yes	11 (7.24%)	5 (10.00%)	
Old pulmonary tuberculosis			0.252
No	146 (96.05%)	46 (92.00%)	
Yes	6 (3.95%)	4 (8.00%)	
Interstitial pneumonia			0.317
No	149 (98.03%)	50 (100.00%)	
Yes	3 (1.97%)	0 (0.00%)	
Tumor			0.721
No	144 (94.74%)	48 (96.00%)	
Yes	8 (5.26%)	2 (4.00%)	
Other underlying pulmonary diseases			0.114
No	130 (85.53%)	47 (94.00%)	
Yes	22 (14.47%)	3 (6.00%)	
Immune deficiency/disorder			0.857
No	145 (95.39%)	48 (96.00%)	
Yes	7 (4.61%)	2 (4.00%)	
No complications			0.093
No	66 (43.42%)	15 (30.00%)	
Yes	86 (56.58%)	35 (70.00%)	
Lymph node calcification			0.661
No	130 (85.53%)	44 (88.00%)	
Yes	22 (14.47%)	6 (12.00%)	
Pleural thickening			0.121
No	80 (52.63%)	20 (40.00%)	
Yes	72 (47.37%)	30 (60.00%)	

PTB, pulmonary tuberculosis; TB,tuberculosis; CRP, C-reactive protein; PCT, procalcitonin; ESR, erythrocyte sedimentation rate.

**Table 2:** Baseline characteristics of 5 initially missed PTB cases.

No.	Gender	Age	Symptoms	Comorbidities	Lesion location
1	Female	69	Asymptomatic	nope	Minimal exudative changes in RUL and RML
2	Female	57	Chronic cough	Bronchiectasis, Interstitial lung disease	Bilateral proliferative foci
3	Female	67	Cough with hemoptysis	Interstitial lung disease	Minimal exudative changes in RUL, RLL and LLL
4	Male	74	Cough with hemoptysis	Bronchiectasis	Bilateral minimal exudative changes, Cavitary lesions in RUL and LLL
5	Female	65	Cough with hemoptysis	Bronchiectasis	Minimal exudative changes in LUL, RML and RLL

PTB, pulmonary tuberculosis; RUL, Right Upper Lobe; RML, Right Middle Lobe; RLL, Right Lower Lobe; LUL, Left Upper Lobe; LLL, Left Lower Lobe.

### 3.2 Diagnostic ability of radiologists with different experience levels in initial TB diagnosis

**Figure 2:**
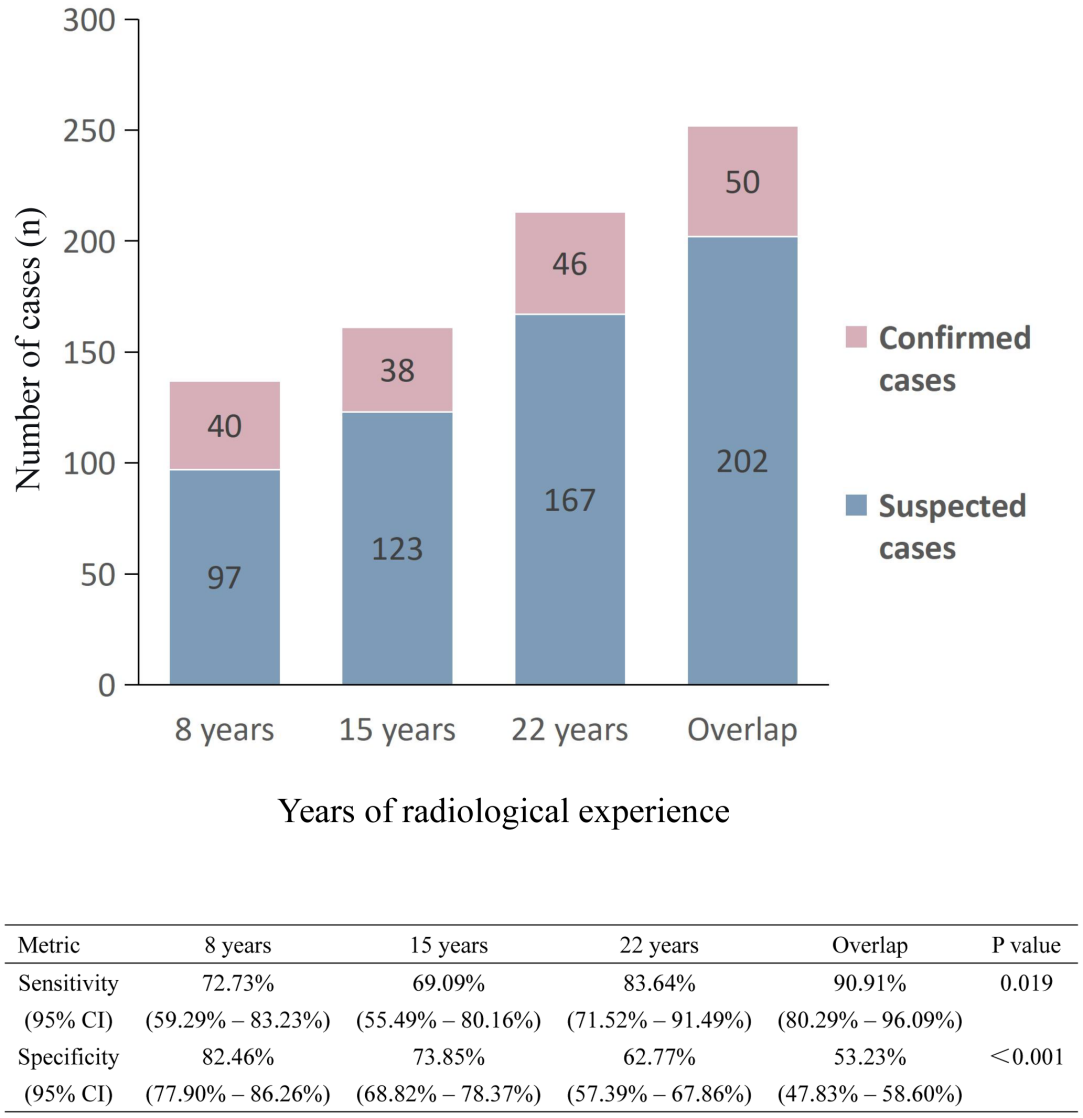
Diagnostic performance of radiologists with different experience in evaluating PTB.

We compared the diagnostic performance of radiologists with 8, 15, and 22 years of experience in diagnosing initial PTB cases (Figure 2). The results showed that as radiologists’ experience increased, the PTB missed diagnosis rate decreased. The combined diagnosis by three radiologists could further minimize the missed diagnosis rate (27.27% [95% CI: 16.77–40.70%] vs. 30.91% [95% CI: 19.73–44.51%] vs. 16.36% [95% CI: 8.38–29.82%] vs. 9.09% [95% CI: 3.80–19.84%], *p* = 0.019). The missed diagnosis numbers under the four scenarios were 15, 17, 9, and 5, respectively. Notably, as sensitivity improved (72.73% [95% CI: 59.29–83.23%] vs. 69.09% [95% CI: 55.49–80.16%] vs. 83.64% [95% CI: 71.52–91.49%] vs. 90.91% [95% CI: 80.29–96.09%], *p* = 0.019), specificity declined (82.46% [95% CI: 77.90–86.26%] vs. 73.85% [95% CI: 68.82–78.37%] vs. 62.77% [95% CI: 57.39–67.86%] vs. 53.23% [95% CI: 47.83–58.60%], *p* <0.001). A sensitivity analysis evaluating a more stringent definition (≥2 radiologists in agreement) yielded sensitivities of 61.82–69.09% and specificities of 82.15–86.46% (Table 3).

**Table 3:** Sensitivity analysis of diagnostic performance when requiring agreement by at least two radiologists for suspected pulmonary tuberculosis.

Metric	8-year + 15-year	15-year + 22-year	8-year + 22-year
Sensitivity (95% CI)	61.82% (48.62–73.51%)	63.64% (50.37–75.12%)	69.09% (55.99–79.74%)
Specificity (95% CI)	85.23% (81.01–88.71%)	82.15% (77.56–85.92%)	86.46% (82.34–89.82%)

### 3.3 Comparison of NTS and CMTs in PTB diagnosis

**Figure 3:**
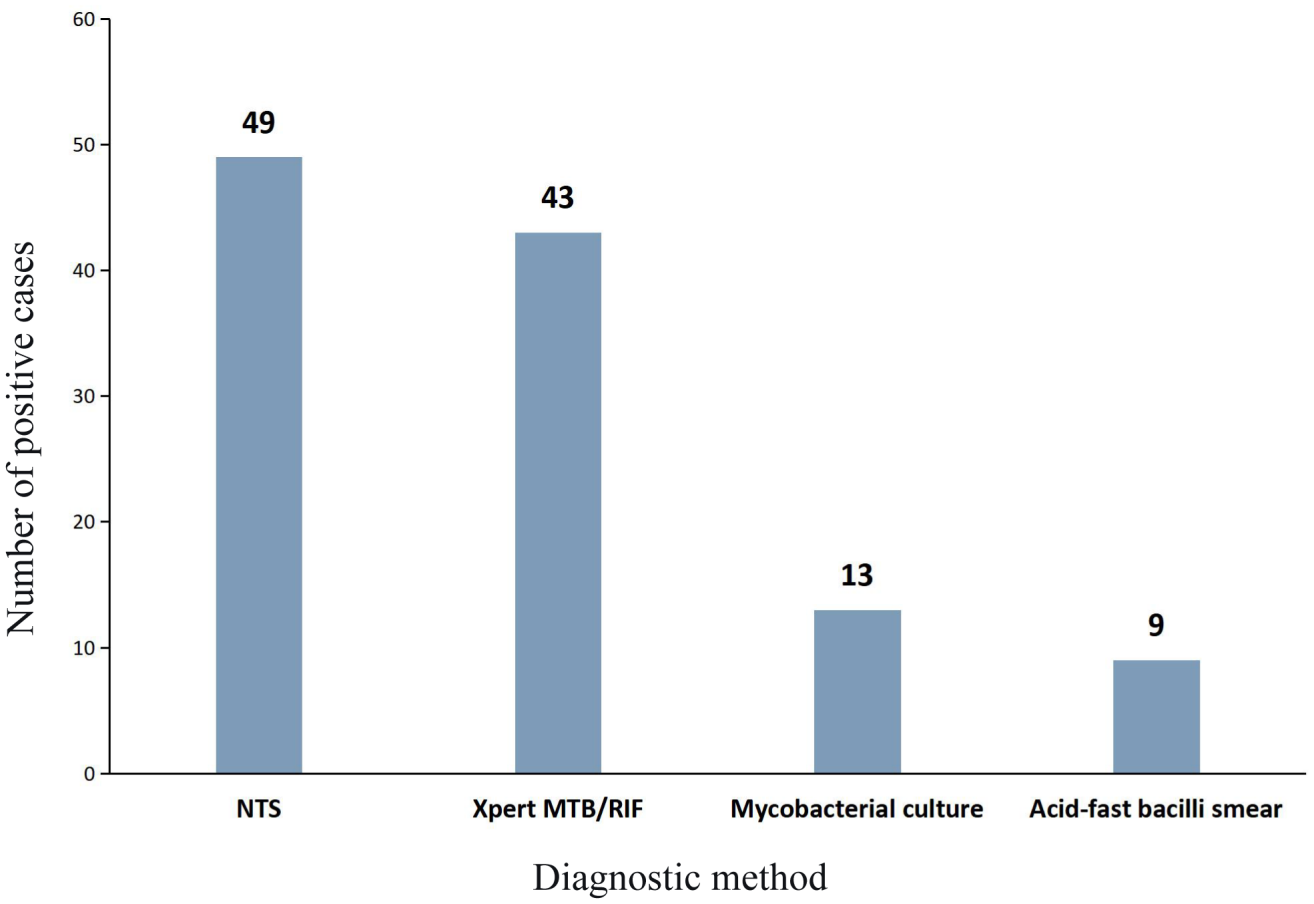
Comparison of NTS and CMTs in PTB diagnosis.

**Figure 4:**
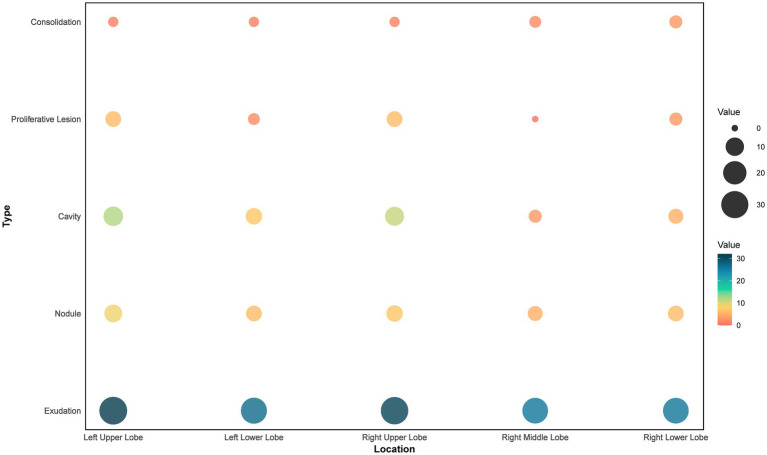
Lung lesion bubble chart.

Among the confirmed cases of PTB, we compared the diagnostic performance of NTS with that of CMTs for tuberculosis (Figure 3). NTS demonstrated superior performance (NTS: 49 cases vs. Xpert MTB/RIF: 43 cases vs. AFB: 9 cases vs. Mycobacterial culture: 13 cases). The sensitivity of NTS was higher than Xpert MTB/RIF, mycobacterial culture, and acid-fast bacilli smear (89.09% [95% CI: 78.17–95.02%] vs. 78.18% [95% CI: 65.44–87.12%] vs. 23.64% [95% CI: 13.99–36.99%] vs. 16.36% [95% CI: 8.38–28.82%], *p* < 0.001). Additionally, we summarized the chest CT imaging features of all 55 TB patients, finding that exudation was the most common pulmonary manifestation, followed by nodules and cavities. The left upper lobe and right upper lobe were the most frequently affected sites. A bubble chart was generated to illustrate all imaging findings (Figure 4).

### 3.4 Exploration of NTS combined with CMTs to improve PTB diagnosis

**Figure 5:**
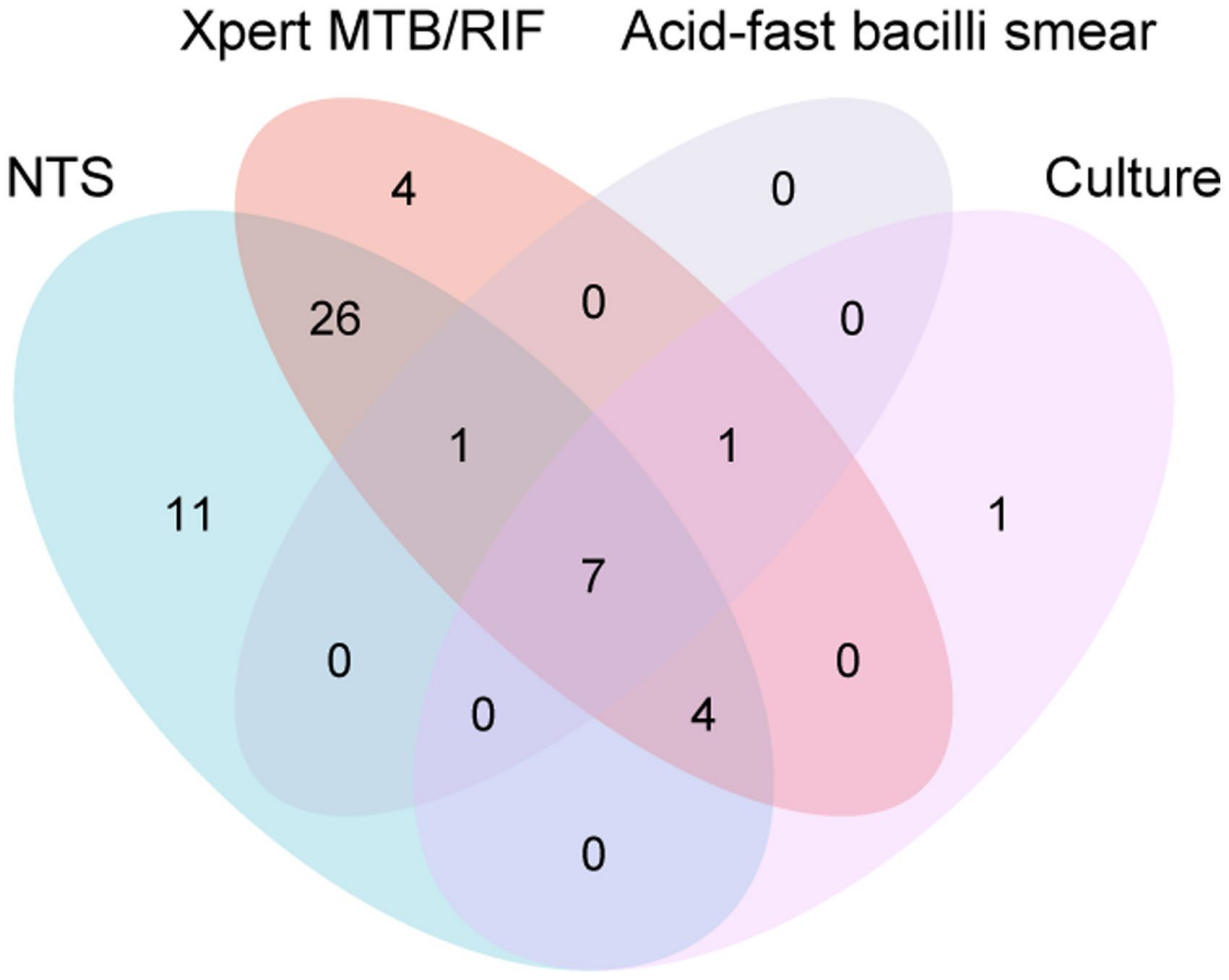
Venn diagram of positive tests for patients of pulmonary tuberculosis.

**Figure 6:**
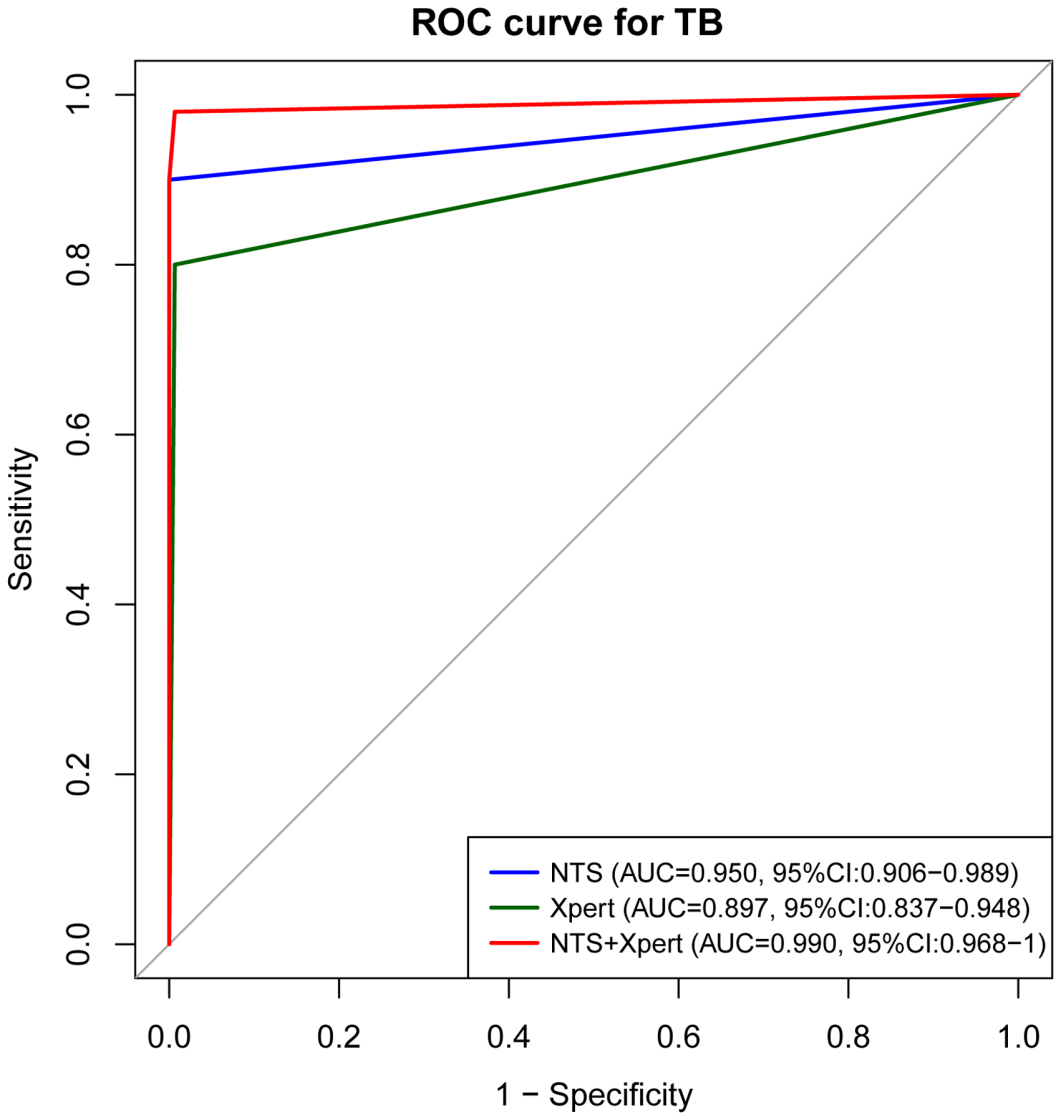
ROC curve analysis.

A Venn diagram was created to depict the detection of MTB using different diagnostic methods (Figure 5). Furthermore, we conducted ROC curve analysis for Xpert MTB/RIF, NTS, and their combination (Figure 6). The AUC values were 0.990 (95% CI: 0.968–1.000) for NTS combined with Xpert MTB/RIF, 0.950(95% CI: 0.906–0.989) for NTS alone, and 0.897(95% CI: 0.837–0.948) for Xpert MTB/RIF alone.

## 4 Discussion

Tuberculosis typically presents with nonspecific symptoms. Currently, delays in seeking medical care and the inefficiency of healthcare systems in identifying TB patients are major contributors to China’s high TB burden (19). Delays in healthcare-seeking, diagnosis, and treatment initiation are common among PTB patients in both high- and low-TB-burden regions globally (20, 21). In low- and middle-income countries, up to 42% of patients exhibit delayed healthcare-seeking behavior after developing TB-related symptoms (22), while in China, over 50% of symptomatic PTB patients fail to seek timely medical attention (23). This underscores that early diagnosis of pulmonary TB within healthcare systems depends both on patients’ timely healthcare-seeking behavior and on enhancements in imaging and clinical diagnostic capabilities to minimize missed diagnoses.

In our study, we compared confirmed and excluded PTB cases among patients with suspected PTB and found that newly diagnosed PTB patients shared similar baseline characteristics with non-TB patients. There were no statistically significant differences in symptoms, inflammatory biomarkers, or comorbidities between the two groups, except for age. Given the current uncertainties in symptom-based and laboratory-based diagnosis, clinicians face significant challenges in identifying PTB. Additionally, studies have shown that radiologists’ experience is crucial for early PTB diagnosis (7). While emerging techniques like radiomics are being explored to enhance diagnostic accuracy (24), low-income countries and primary healthcare facilities still heavily rely on skilled radiologists. Our analysis of interobserver agreement showed that the two senior radiologists had higher concordance in identifying PTB-related CT findings, while the less-experienced radiologist showed only moderate agreement with either senior. To prioritize screening sensitivity, we adopted a combined-suspicion strategy (≥1 radiologist positive), which increased sensitivity to 90.91% (95% CI: 80.29–96.09%) but reduced specificity to 53.23% (95% CI: 47.83–58.60%). Although this may raise concerns about false-positive imaging flags, chest CT serves only as a preliminary screening tool in our diagnostic pathway. All suspected cases are subsequently tested with NTS and CMTs, which provide very high specificity (AUC for NTS plus Xpert reached 0.990 [95% CI: 0.968–1.000]). This approach ensures that very few true TB cases are missed at the screening stage while molecular confirmation effectively rules out false positives. Thus, the loss in imaging specificity is well-compensated by the downstream high-specificity assays and is clinically acceptable in TB control.

Furthermore, we performed a sensitivity analysis to assess the impact of adopting a more stringent ‘suspected TB’ definition, i.e., requiring agreement by at least two radiologists. As shown in Table 3, any two-reader consensus combination resulted in a marked decline in sensitivity, with values ranging from 61.82 to 69.09% (average 64.85%), compared to 90.91% (95% CI: 80.29–96.09%) achieved by the ‘at least one radiologist’ criterion. While specificity improved to 82.15–86.46%, over one-third of true PTB cases would have been missed at the initial screening stage under the stricter definition. Given that 75.25% of the patients in our suspected TB group presented with at least one cardinal respiratory symptom (Table 1), the pretest probability was already elevated, and missing a case at this stage would carry substantial public health risk, including continued transmission and delayed treatment initiation (25, 26). Therefore, we deliberately chose the broad screening definition to maximize sensitivity, relying on the subsequent high-specificity molecular confirmation (AUC = 0.990 for NTS plus Xpert MTB/RIF) to efficiently resolve false-positive imaging flags. This two-tier strategy—broad clinical-radiological screening followed by precise molecular diagnosis—is both clinically justified and epidemiologically prudent, particularly in high-burden settings where active case finding is a cornerstone of TB control (27, 28).

At our institution, the radiology department maintains a well-balanced team consisting of 8 junior radiologists (<10 years of experience), 13 mid-level radiologists (10–20 years), and 12 senior radiologists (>20 years). This distribution ensures sufficient expertise for TB imaging screening and allows hierarchical consultation. Furthermore, the proposed screening model can be extended to settings with fewer senior radiologists through tele-radiology networks and dedicated tuberculosis imaging training. Our data suggest that even a single trained radiologist can achieve a sensitivity of 83.64% (95% CI: 71.52–91.49%), and the addition of molecular confirmation provides a robust safety net.

The chest imaging manifestations of secondary PTB are highly diverse. Mild cases may present with patchy shadows, nodules, or linear opacities, while others may exhibit tuberculomas or isolated cavities. Severe cases can manifest as lobar infiltrates, caseous pneumonia, multiple cavities, or bronchogenic dissemination. Chronic or recurrent cases may lead to destroyed lungs, characterized by volume loss, fibrotic thick-walled cavities, bronchiectasis, or calcifications (15). Traditionally, secondary PTB predominantly affects the apical and posterior segments of the upper lobes and the dorsal segments of the lower lobes, with lesions typically appearing as nodular or branching linear structures, bronchial wall thickening, ill-defined nodules, cavities, or lobular consolidations (29, 30). Our analysis of chest CT findings in 55 confirmed PTB patients confirmed that the upper lung apices remain the most common sites of involvement, consistent with prior studies. Nodules and cavities were frequent manifestations, though exudative changes were the most predominant. Among the five cases initially missed by radiologists, four had concurrent pulmonary complications such as bronchiectasis or interstitial lung disease, suggesting that pre-existing structural abnormalities may complicate diagnosis. Further large-scale studies are needed to refine diagnostic criteria in such cases.

NTS is an emerging third-generation sequencing technology known for its rapid sequencing capability, enabling long-read sequencing of tens to hundreds of kilobases (31). Additionally, it allows real-time data analysis, making it valuable for infectious disease surveillance and genomic research (32, 33). Its strength in identifying GC-rich regions and base modifications in MTB sequences has led to its increasing application in bacterial detection (34, 35). In our study, NTS was compared with CMTs for MTB detection, and it demonstrated significantly superior performance over Xpert, culture, and AFB, consistent with findings from previous studies (35). Notably, NTS requires considerably less time for both experimental procedures and result reporting than CMTs, mNGS, and alternative methods. In addition, in China, the cost of NTS is under 800 RMB (≈100 USD), just a quarter of that of mNGS. Moreover, NTS has even been utilized for detecting MTB drug resistance (36, 37). Notarte and colleagues showed that targeted sequencing can achieve clinically meaningful mycobacterial identification and resistance detection (98.8% concordance for NTM, with subspecies resolution), directly reinforcing the rationale for our NTS-based diagnostic approach to tuberculosis (38). We further explored combining NTS with conventional microbiological tests (CMTs) to improve TB diagnosis. ROC curve analysis revealed that the addition of NTS substantially enhanced diagnostic accuracy, consistent with a recent meta-analysis of nanopore sequencing for tuberculosis, which reported a pooled sensitivity of 86% and specificity of 95% across 32 studies (39). Given its low per-test cost, short turnaround time, and centralized batch-testing feasibility, NTS already helps mitigate the economic impact of false-positive screens; a formal cost-effectiveness analysis would nevertheless be valuable to quantify this balance and further support clinical implementation.

In 2014, the WHO launched the End TB Strategy (28). However, the global TB incidence rate has declined by only ~2% annually in recent years, with an 11% cumulative reduction from 2015 to 2020 (40). Inadequate case detection and diagnostic limitations remain key barriers to achieving targets. The WHO estimates that over 40% of TB cases went undiagnosed or unreported in 2020 (41). Some studies have suggested that treatment-naïve TB patients are at higher risk of diagnostic delays compared to retreatment cases (42), possibly due to the latter’s prior experience with TB screening and greater disease awareness, which may prompt earlier healthcare-seeking behavior (43). Our study demonstrates that radiologist-led imaging screening reduces initial missed diagnoses, while NTS combined with CMTs enhances detection in new TB cases. This approach could significantly curb TB underdiagnosis and transmission.

As a retrospective study, our research has several constraints. First, the small number of radiologists limits the generalizability of our imaging diagnostic findings. Second, NTS was evaluated against a reference standard that included its own results. This design inherently overestimates diagnostic performance (particularly sensitivity and AUC). Thus, the reported estimates are optimistic. Third, although the findings support NTS’s diagnostic advantages, the limited sample size of confirmed PTB cases introduces statistical uncertainty. Therefore, future prospective studies with larger, more diverse, multicenter cohorts and improved study designs are necessary to validate the diagnostic pathway proposed here.

## 5 Conclusion

TB prevention and control require overcoming the dual challenges of missed diagnoses at initial presentation and insufficient diagnostic technologies. This study proposes a dual-pathway strategy: enhancing radiological expertise through specialist consultations to reduce missed diagnoses in treatment-naïve patients, and combining NTS with conventional diagnostic methods to significantly improve *Mycobacterium tuberculosis* detection. Within the limitations of our retrospective, single-center design (including potential incorporation bias), the combined use of NTS and Xpert MTB/RIF showed promising diagnostic accuracy (AUC 0.990 [95% CI:0.968–1.000]). These findings suggest a feasible approach for early TB diagnosis, but prospective multicenter studies with independent reference standards are needed for confirmation.

## Data Availability

The raw sequence data reported in this paper have been deposited in the Genome Sequence Archive (Genomics, Proteomics & Bioinformatics 2025) in National Genomics Data Center (Nucleic Acids Res 2025), China National Center for Bioinformation / Beijing Institute of Genomics, Chinese Academy of Sciences (GSA-Human: HRA019488) that are publicly accessible at https://ngdc.cncb.ac.cn/gsa-human.
