# Supplementary material for: Clinical application of chest CT image screening combined with nanopore targeted sequencing in improving the diagnosis rate of pulmonary tuberculosis patients
Source: Front Public Health. 2026 Jul 23;14:1826208. doi: 10.3389/fpubh.2026.1826208 (PMC13442664; doi:10.3389/fpubh.2026.1826208)
Supplement: Supplementary file 1 [file Data_Sheet_1.pdf]

Supplementary 1，Detailed procedure of NTS testing

NTS testing: BALF samples (>5mL, placed in sterile sputum containers) are collected and immediately transported to a commercial laboratory (Hangzhou Dean Medical Laboratory (Hangzhou, China) using dry ice for testing.

Table 1. Instruments and equipment used

Instrument name	*Model*	*Manufacturer and Producer*
Desktop refrigerated centrifuge	*BY-R320*	*Baiyang, Beijing*
Vortex Mixers	*QL-901*	*Qilinbeire, Haimen*
High speed centrifuge	*BY-G1200*	*Baiyang, Beijing*
Tissue grinding homogenizer	*TL2020S*	*Dinghaoyuan, Tianjing*
Fluorescence quantizer	*qubit 4.0*	*Thermo Fisher*
Constant temperature water bath	*DKT-200*	*Miou, Hangzhou*
PCR instrument	*Hema9600*	*Heima, Zhuhai*
Nanopore sequencer	*GridION MK1*	*Oxford Nanoporous Technology*

1. Sample Preprocessing

Liquefaction of alveolar lavage fluid (only for viscous samples), add a certain amount of liquefied fluid and 20% by volume 1:1 μ L XC, shake and mix well, shake at 1000 rpm on a constant temperature mixer for 1-3 minutes, incubate at 37 ℃ for 10 minutes (if the liquefaction is still viscous, increase the amount of liquefaction liquid appropriately for secondary liquefaction), take 1mL (the maximum volume of less than 1mL) and add it to a 1.5mL sterile centrifuge

tube, centrifuge at 12000 rpm for 2 minutes, and discard 950 μ L supernatant (residual liquid approximately 30-40 μ L) . Add 200 μ L cracking solution LS, shake or blow with a gun head to suspend and disperse the precipitate, and react at 37 ℃ for 10 minutes. Add 100mg of glass beads and shake the wall breaker at 2000rpm for 8 minutes (pausing for 30 seconds every 1 minute) at high speed.

2. Nucleic acid extraction

Add 400 μ L cracking liquid LE, 20 μ L Proteinase K, blow and mix well, and absorb about 600 after brief centrifugation μ Add L supernatant to the prepared 96-well deep well plate in the 1st/7th row of holes, and place the 96-well plate and magnetic sleeve into the Hema nucleic acid automatic extractor according to the requirements of the instrument manual, and automatically run according to the set program. After the program is completed, transfer the nucleic acid solution from the 6/12 well to a 1.5 mL EP tube and measure the DNA concentration using a Qubit 3.0 fluorescence quantitative analyzer.

3. Library construction

3.1 Targeted amplification

Prepare a reverse transcription reaction mixture in a 0.2 mL PCR centrifuge tube according to the ratio in Table 2 (the reaction solution needs to be prepared and packaged on an ice box, and the ice box needs to be kept at low temperature), mix it gently, centrifuge briefly, and place it in a PCR machine for reverse transcription reaction. The reaction conditions are as

follows: 37 ℃ for 15 minutes, 85 ℃ for 5 seconds, 4 ℃ hold。

Table 2. Reverse transcription reaction mixture

Component	*Per sample/volume（μL)*
RNA	*8*
Reverse transcription reaction fluid	*2*
Total	*10*

Prepare the required amount of PCR reaction mixture in a new centrifuge tube as shown in Table 3, and perform the PCR reaction according to the reaction conditions shown in Table 4 after completion.

Table 3. PCR Reaction System

Component	*Per sample/volume（μL)*
Reverse transcript product	*4μl*
PCR amplification reaction solution	*36μl*
Total	*40 μl*

Table 4 PCR Reaction Procedure

Temperature	*Time*	*Cycle*
95℃	*2 min*	*1*
95℃	*15 s*	*35*
60℃	*2min 15 s*	

72℃	5 min	1
4℃	Hold	1

After the reaction is completed, add 1.5X volume of magnetic beads for purification, blow and mix well, and let stand for 5 minutes; Transfer to a magnetic rack, wait for the solution to clear (about 2 minutes), discard the supernatant, and use 150 μ Wash twice with 70% -80% ethanol (do not disturb the magnetic beads), immediately remove and place the remaining ethanol on a magnetic rack with 10 μ L Pipette absorbs the solution, leaves it to dry for 2min, and finally washes it with 25 μ L ddH2O water at room temperature for 5min. Place it on the magnetic rack, and after the solution is clarified (about 2min), suck out the eluent for standby and measure the concentration.

3.2 DNA fragment end repair and purification

Prepare the final reaction solution according to Table 6 in a 0.2ml PCR tube, gently stir and mix, 20 ℃ for 5 minutes, 65 ℃ for 5 minutes, 4 ℃ hold

Table 6 DNA fragment end repair and purification System

Reagent	Volume
H2O	Calculate the volume of added DNA and replenish it with water to 12.5ul
DNA	
Terminal modifying Buffer	1.75μl

Terminal modifying enzyme	*0.75μl*
Total	*15 μl*

Prepare reaction solution according to Table 7, gently stir and mix, 20 ℃ for 20 minutes, 65 ℃ for 10 minutes, 4 ℃ hold；

Table 7. Barcode Connection Reaction System

Reagent	*Volume*
H2O	*2.5 μl*
erminal modification products	*5 μl*
Native Barcode	*2.5 μl*
Connect the reaction liquid	*10 μl*
Total	*20 μl*

*After connecting Barcode, mix all the samples into one tube. Add 1.5 * magnetic beads to the sample tube, blow and mix well, and let it stand at room temperature for 10 minutes (gently stir and mix 2-3 times with your fingers); After the rest is completed, immediately detach and transfer to a magnetic rack. After the solution clarifies (about 5 minutes), discard the supernatant; Remove the sample tube from the magnetic holder and clean it twice with 1.5 * SFB. After each addition of SFB, it should be thoroughly mixed with the magnetic beads, then immediately separated and placed on the magnetic holder. After clarification, discard the liquid. Add 100 μ l of 80% ethanol (do not disturb the magnetic beads), immediately dissociate, remove the remaining ethanol, and*

dry for 30 seconds; Add 35 μ l of water, blow and mix well, let stand at room temperature for 5 minutes, transfer immediately to a magnetic rack, wait for the solution to clear, transfer for backup, and measure the concentration.

3.3 Adapter Mix (AMX) Connection Reaction and Purification

Prepare the reaction solution according to Table 8, gently stir and mix, and react at 20 ℃ for 20 minutes；

Table 8. Adapter Connection Reaction System

Reagent	Volume
Pooled barcoded sample	30 μl
Adapter Mix II (AMII)	5 μl
Ligation buffer	10 μl
ligase	5 μl
Total	50 μl

Add 75 to the connecting product after the reaction is completed μ Purify with L magnetic beads, blow and mix well, let stand for 10 minutes, and use 125 μ Clean the L SFB twice, mix it thoroughly with the magnetic beads after each addition of SFB, and then place it on a magnetic rack after instant separation. After clarification, discard the liquid. Immediately after separation, place it on a magnetic rack and apply 10% residual SFB μ L Pipette shall be sucked clean and dried after standing for 30s; Join 12 μ L EB, blow and mix well, let stand for

5 minutes, transfer to a magnetic rack after instant separation, and wait for the solution to clear before measuring the concentration and backup.

4. Machine sequencing

Melt Sequencing Buffer (SQB), Loading Beads (LB), Flush Tether (FLT), and a tube of Flush buffer (FB) at room temperature. Prepare priming mix in a 1.5 mL centrifuge tube: Take 1 FB and add 30 μ L FLT, shake well and centrifuge briefly. Open the cover of MinION Mk1B and load the chip. Slide the sampling port cover clockwise, open the chip Priming port, set the 1mL Pipette to 180 μ L, insert the suction head of the Pipette into the Priming port, slowly rotate the rotary wheel counterclockwise to make the dial display 200 μ L, or until a small amount of buffer can be seen entering the suction head, and then draw 800 μ The priming mix of L is slowly injected into the flow cell from the Priming port to avoid bubbles, and it is allowed to stand at room temperature for 5 min. Fully mix the loading beads (LB) with a Pipette. In the new EP tube, prepare the sample library as follows: 37.5 μ L Sequencing Buffer (SQB), 25.5 μ L LB, 40-80 ng DNA library, fill with water to 75 μ L, and perform sequencing on the machine.

5. Bioinformatics analysis

The format of the original data file generated by MinION sequencer is fast5. The MinKnow software is used to complete real-time identification and generate the fastq file, and the MinKnow software is used to filter low quality value sequences（10M）. The filtered data was subjected to host DNA removal

using Minimap2 2.17-r941(1) software (using the human genome reference sequence Hg38). Multi Sequence alignment of sequencing data and determination of pathogenic microorganisms After data filtering and de host DNA sequencing data, Centrifuge(2) v1.0.3 is used for multi Sequence alignment with NCBI non redundant nucleic acid data (NT) library.

Supplementary 1

Table 1. 354 respiratory pathogens were included in the panel of this study

Bacterium (n=236)	*Gram-positive bacillus*	*Mycobacterium tuberculosis complex group (Mycobacterium tuberculosis, Mycobacterium bovis, Mycobacterium Africana), Mycobacterium leprae, nontuberculous mycobacterium (Mycobacterium avium, Mycobacterium intracellular, Mycobacterium abscess, Mycobacterium Kansus, Mycobacterium Malmo, Mycobacterium toad, Strumfulosa, Mycobacterium chelonis, Mycobacterium fortuitus, Mycobacterium marinum, Mycobacterium gordonum) Bacillus, Mycobacterium myxigenes, Mycobacterium ulcerans, Mycobacterium flavus, Mycobacterium Marseille, Mycobacterium Kumamoto, Mycobacterium Haemophilus, Mycobacterium paracytosus, Mycobacterium Genevae, Mycobacterium Asiatica, Mycobacterium Bohemia, Mycobacterium carinii, Mycobacterium hiddeni, Mycobacterium simiae, Mycobacterium Stefani, Mycobacterium triplica, Mycobacterium bacteremia, Mycobacterium neoaureus, Mycobacterium stigmatis, Mycobacterium Colombia), Nocardia in pus, Nocardia abscess, Nocardia Wallace, star, Nocardia Brasiliensis, Nocardia otitis of guinea pig, Nocardia dermatitis, Nocardia pseudobrasiliensis,*

		Nocardia neocardia, Transsylvania, Nocardia veteranum, Nocardia galsenkirchen, Nocardia brachystreptococcus, Clostridium percapsulatus, Lysogroup Clostridium difficile, Clostridium tetanus, Clostridium botulinum, Clostridium difficile, Clostridium Northrix, Erysipelothrix erysipelogenes, Listeria monocytogenes, Whipples' trophia, Deficient bacteria, Actinomyces madurae, Actinomyces actinoides, Actinomyces caries, Streptomyces, Bacillus anthracis, Bacillus cereus, Clostridium difficile, Clostridium septicus Bacillus, Arcanobacterium pyogenes, Arcanobacterium haemolyticus, Propionibacterium pemphigi, Corynebacterium McCorilli, Corynebacterium diphtheriae, Corynebacterium striatum, Corynebacterium pseudotuberculosis, Rhodococcus equi, Rhodococcus rhodococcus fuchsia
	Gram-positive coccus	*Enterococcus faecalis, Enterococcus faecium, Enterococcus aviae, Enterococcus gallinaceus, Enterococcus plumboflavus, Streptococcus agalactis, Streptococcus galactis, Streptococcus pneumoniae, Streptococcus pyogenes, Streptococcus suis, Streptococcus bradystis, Streptococcus cristae, Streptococcus constellatus, anaerobic peptococcus, Streptococcus Donae, Streptococcus gallicae, Streptococcus equi, Streptococcus Miller, Streptococcus bovis, Streptococcus Canis, Angina Streptococcus, Streptococcus ovis, Streptococcus intermedius, Streptococcus piggut, Streptococcus equi,*

		Streptococcus infantile, Streptococcus parahaematococcus, Streptococcus oral, Staphylococcus aureus, Staphylococcus epidermidis, Staphylococcus Luteus, Staphylococcus hominis, Staphylococcus arletus, Staphylococcus chromogenes, Staphylococcus auris, Staphylococcus saprophyticus, Staphylococcus slowus, Staphylococcus pseudointermedius Staphylococcus cocci, Staphylococcus Koch, Staphylococcus Kerri, Staphylococcus equi, Staphylococcus imitatus, Staphylococcus xylosus, Staphylococcus haemolyticus, Staphylococcus goat, Staphylococcus Schisteri, Staphylococcus Sciuris, Staphylococcus capitans, Staphylococcus Woweri, Staphylococcus vealis, Staphylococcus intermedius, Staphylococcus suis, Twinus morbiae, Micrococcus luteus, Cookella roseus, Staphylococcus Streptomyces granulosus and Rhodotella mucilaris
	Gram-negative bacilli	*Acinetobacter Baumannii, Acinetobacter Pitter, Acinetobacter calcic Acetate, Enterobacter sakazakii, Enterobacter cloacae, Proteus mirabilis, Bacillus aureus indolicogenes, Bacillus alcaligenes faecalis, Citrobacter Freudi, Corynebacterium jeri, Bacillus cardiobacterium humani, Bacillus pulmonoides, Bacillus palustris humani, Bacillus rigovitogeni, Bacillus pestis, Bacillus brucei, Bacillus fluoriformis Bozmanni Citrobacter monciformis, Agnetocosa, Citrobacter kirkii, Streptococcus candidiformis, Campylobacter jejuni, Campylobacter foetus, Corynebacterium ureae, Proteus vulgaris, Bordetella pertussis, Bordetella parappertussis, Bordetella avium, Bordetella houthi,*

		Burkholderia melanoides, Burkholderia melanoides, Burkholderia acoris, polyphagosis Burkholderia, Klebsiella aerogenes, Klebsiella acidogenes, Klebsiella pneumoniae, Klebsiella mutans, Escherichia coli, Haemophilus influenzae, Haemophilus parainfluenzae, Haemophilus Haemolyticus, Legionella pneumophila, Pseudomonas aeruginosa, Pseudomonas putidis, Serratia marcescens, Stenotrophomonas maltophilia, Bacillus morgana, Salmonella enterica, Elizabethia meningosepticum , Francisella tulafarensis, Serratia liquefaciens, Pasteurella multocida, Pseudomonas alcaligenes, Kingella kimi, Aeromonas hydrophila, Shigella Sonnei, Bartonella veneri, Bartonella baculosa, Bartonella elizabethica, Elizabethica pacifica, alcaligenes Providencia, Panthenia globosa, Edwardsiella obliqua, Prevotella diplex, Hafniella apiaria, Shiwanella alginae, Vibrio vulnificus, Vibrio cholerae, Rolstonia mannitolytica, Raoulia ornitholytica, Avenella Americana, Rolstonia pieteri, Pseudomonas oryzae, Aikenella erotica, Cynocytophilia Canis, Salmonella typhi, Procencia Steinii, Yersinia enterocolitica

	Gram-negative coccus	*Neisseria meningitidis, Neisseria gonorrhoeae, Neisseria microflava, Neisseria desiccata, Moraxella katamensis, Moraxella Oslo, Moraxella lacunaris*
	Aanaerobe	*Bacteroides fragilis, Clostridium nucleatum, Clostridium periodontidis, Clostridium necrosis, Prevotella, Actinomyces, Peptostreptococcus, Peptococcus nigra, Prevotella griseus, Porphyromonas gingivalis, Forsestanella, Veillonococcus atypicus, Veillonococcus parvus, Peptonophilus lacrimalis*
Fungi（n=33）		*Aspergillus aflatus, Aspergillus oryzae, Aspergillus fumigatus, Aspergillus nidus, Aspergillus Niger, Aspergillus terreus, Candida albicans, Candida auris, Candida glabrata, Candida Tropicalis, Candida Portuguese, Pichia Kudriazweil (Candida kronensis） Bacillus, Rhizopus oryzae, Sedospora apicum, Fusarium, Blastomyces dermatitis, Sporotrichum Schenckii*
Others (n=18)	*Mycoplasmas*	*Mycoplasma pneumoniae, Mycoplasma hominis, ureaplasma parvos, Ureaplasma urealyticum*
	Chlamydia	*Chlamydia pneumoniae, Chlamydia psittaci, Chlamydia trachomatis*
	Spirochaeta	*Syphilis, leptospira*

	Rickettsiae	*Rickettsia plati, Rickettsia Mosii, Rickettsia Rickettsia Rickettsia feline, Rickettsia typhus, Orientia tsutschiggers, Benacosia, Hansebartonia, anaplasmosis granulocyticus*
Viruses (n=58)	*DNA viruses*	*Adenovirus, human adenovirus type B7, human adenovirus type B55, human adenovirus type C1, human adenovirus type C2, human adenovirus type C5, human adenovirus type E4, human adenovirus type B3, human Boca virus, human Parvovirus B19, herpes simplex virus type 1, herpes simplex virus type 2, varicella zoster virus, EB virus (blister type 4) Herpes), Cytomegalovirus (herpes type 5), human herpesvirus type 6, human herpesvirus type 7, human herpesvirus type 8, herpes virus type B, BK polyoma virus, JC polyoma virus, WU polyoma virus, human polyoma virus type 7, KI polyoma virus, human polyoma virus type 5, MW polyoma virus*
	RNA viruses	*Influenza A virus H1N1, influenza A virus H3N2, Avian influenza H5N1, Avian influenza H7N9, influenza A virus (universal), influenza B virus, influenza C virus, parainfluenza virus type 1, parainfluenza virus type 2, parainfluenza virus type 3, parainfluenza virus type 4,*

		human metapneumovirus, coronavirus 229E, coronavirus HK U1, Coronavirus NL63, coronavirus OC43, respiratory syncytial Virus A, respiratory syncytial virus B, human rhinovirus, measles virus, rubella virus, mumps virus, Enterovirus 71, Enterovirus C, Enterovirus D, Coxsackie virus A6, Coxsackie virus A16, Coxsackie virus A21, Coxsackie virus B, Echovirus, Rotavirus, novel coronavirus
Parasites (n=9)		*Paragonimus westermani, Entamoeba histolytica, Echinococcus, Toxoplasma gondii, Microsporidia, Cryptosporidium, Strongyloides stercoralis, Brugia malayi, Toxocara canis*

1. Li H. 2018. Minimap2: pairwise alignment for nucleotide sequences. Bioinformatics 34:3094-3100.
2. Kim D, Song L, Breitwieser FP, Salzberg SL. 2016. Centrifuge: rapid and sensitive classification of metagenomic sequences. Genome Res 26:1721-1729.
